# What Psychology Means To Me

**DOI:** 10.4103/0973-1229.27604

**Published:** 2006

**Authors:** Donelson E. Dulany

**Affiliations:** * *University of Illinois, Also Editor, American Journal of Psychology. ddulany@cyrus.psych.uiuc.edu*

**Keywords:** Consciousness, Awareness, Unconscious, Volition, Behaviorism, Cognitivism, Mentalism

## Abstract

What the title of this article means to me after decades on a university faculty is very broad. It would include topics of my research and writing, of my graduate and undergraduate teaching, and of what I read in the area, including papers that have been submitted to me as editor of the American Journal of Psychology. What I can write here focuses on my research and writing and related metatheoretical views, including what I have considered the deeper and more significant questions formulated in philosophy of mind and submitted to empirical investigation in psychology. Of most active concern today, and over the years, are those asking about the roles of consciousness, symbolic representation, and volition in mental activity and action. Is symbolic representation carried out consciously or unconsciously, or both? This paper recognizes the 19^th^ century attempt to establish a science of consciousness, a behavioristic movement that rejected consciousness as being the soul of theology, a cognitive metatheory giving some place to consciousness but emphasizing non-conscious symbolic representation. This summarizes our experimental examinations of my theories of the source of intentional action, of causal reasoning, and of explicit and implicit learning. It also summarizes the overarching mentalistic metatheory I have described.

## Introduction

Asked to write a paper with this title—*What Psychology Means to Me*—so very much comes to mind, naturally coming out of my personal academic history. My research and writing, and much of my teaching in psychology, have focused on some of the grander questions coming out of the philosophy of mind, questions that have been and continue to be submitted to theoretical and experimental examination within psychology: what are the roles of consciousness, symbolic representation, and volition in mental activity and action? And how might scientific methodology be extended to something as complex as consciousness and the mind?

In fact, is consciousness even a legitimate subject matter for science? If so, are our symbolic representations of a world beyond carried by conscious or unconscious mental states—or both? Can conscious states be causal or is that impression only illusory? Answers to these questions have set apart the influential behavioristic and cognitive metatheories in psychology, as well as the mentalistic metatheory I have proposed.

For me an interest in these questions began with a double undergraduate major in psychology and philosophy, then graduate study in psychology, with a year on a Rockefeller project in Philosophy, Language and Symbolism: graduate study that I saw as preparation for the empirical examination of just those deeper questions for the discipline. These are questions that continue to animate a great deal of research and writing in psychology, including my own. Most of my teaching has been graduate teaching, a course in the methodology-philosophy of science for this discipline and a course in consciousness and the non-conscious, courses naturally focused on those larger questions for the discipline. But with decades on a university faculty, psychology has naturally meant somewhat more to me as well, in teaching undergraduate courses in cognitive and social psychology and even a broad introductory course, in the lecture hall for hundreds and on television for thousands. I currently teach Modern Viewpoints in Psychology for senior majors and graduate students, an examination of the views that have shaped the discipline—behaviorism, psychoanalytic theory, and the currently dominant cognitive metatheory—all of which together I refer to as the “grand sweep of ideas”. And of course, psychology conveys a certain meaning for me as I serve as the Editor of the *American Journal of Psychology* (*http://www.press.uillinois.edu/journals/ajp.html*).

But just how has this “grand sweep of ideas” in psychology and those “grander questions” from philosophy influenced my own research and writing? Psychology has a troubling history of diminishing the role of consciousness, and my research and writing have challenged some of those views and offered alternative theories and an overarching metatheory.

## Behaviorism And Challenges

My early research and theorizing (Dulany, 1968), and earlier, challenged central propositions of stimulus-response theory and its underlying behaviorism, at the time a still dominant ideological position that ruled consciousness out of science entirely. As its famous revolutionist John B. Watson (1924, p. 3) had put it, “Behaviorism claims that ‘consciousness’ is neither a definite nor usable concept; that it is merely a word for the ‘soul’ of more ancient times. The old psychology is thus dominated by a kind of subtle religious philosophy”. And so psychologists, if they were to be scientists, could have nothing to do with consciousness and what they mistakenly believed to be entailed: the metaphysical claims that consciousness as soul was non-material and immortal and something outside a deterministic order. The subject matter of the science was to be behaviour exclusively, and the vocabulary of consciousness—“believe”, “desire”, etc.—was redefined so as to refer to covert muscular activity. They even drew a little support—among the more widely read—from a then fashionable Oxbridge philosophy of language. This zealous movement rather effectively overturned early and only preliminary efforts to establish a science of consciousness (e.g. Titchener, 1898; James, 1890).

Nevertheless, we can now recognize that the behaviorists failed to distinguish between metaphysical and theoretical assertions—a fundamental conceptual error. Assertions that consciousness is non-material or immortal, and/or “free” in the sense of indeterminism are metaphysical assertions, and therefore not subject to empirical examination within science as it was known then or is known today. On the other hand, theories in which conscious states are causal may be empirically examined within science—an entirely secular enterprise—leaving aside commitment to metaphysical claims of their ontological status, ultimate fate, or the degree to which determinism holds. Broad and continuing consequences of this conceptual error, even outside behaviorism, are discussed in Dulany (2003). For many, however, determinism and materialism have simply been useful *heuristic* assumptions.

How then did we empirically examine a theory of causal action of conscious states, including a volitional intention, a theory that lies outside behaviorism? On behaviorism's stimulus-response theory, any consequence, rewarding or punishing, selectively strengthens or weakens the response that produces it by “automatic action of reinforcers”—where that means without the intervention of conscious and volitional states. On a theory of conscious control I offered instead (Dulany, 1968), that selection of the response is causally controlled by a network of conscious states each taking a quantitative value—an intention controlled by the product of an outcome rule (awareness of what response the reward or punishment follows) and evaluation of the outcome, together with the product of a social rule (awareness of what others think one should do) and motivation to comply. These are all conscious states to be assessed with phenomenal reports that consisted of marking quantitative scales.

With prior medical approval—and dressed for the occasion—our experimental subjects one by one entered a controlled environment chamber kept at a constant temperature of 110 degrees Fahrenheit and 40% humidity, a chamber that had been used for aeronautics research within our College of Engineering. On each of 100 trials, they picked and read one of two sentences. If they read a sentence with “vanished” in it, a 10 sec stream of air blew across their faces, but not if they selected a sentence with “disappeared”. In one group, the stream of air was 40 degrees cooler, in a second group the same as chamber temperature, and in a third (less fortunate) group 40 degrees hotter—outcomes with subjective values that were rather clearly different and so evaluated on our scales. Crossed orthogonally with this, three sub-groups within each condition were informed either that the stream of air meant they were correct, incorrect, or neither (“just a routine adjustment of temperature in the chamber”).

Although this design permitted examination of a range of hypotheses, the central points were these: for subjects unaware of what sentences the stream of air followed—the outcome rule—there was no evidence of learning to choose or avoid those sentences, despite varying the value of the outcome within wide but humane limits. That meant no evidence for an unconscious process of “automatic action of reinforcers”, of unconscious strengthening or weakening of that response, the fundamental principle for the behaviorists’ S-R learning theory. Furthermore, the quantitative reports of conscious states in response to focused assessments permitted examination of an alternative theory I proposed. Reports of intentions strongly predicted frequency of correct responses, and a linear regression equation strongly predicted reports of conscious intentions from reports of values on variables within the outcome and social components.

This was not only a challenge to S-R theory and behaviorism; it provided empirical support for an alternative theory in which conscious states, including volitional intentions, causally interacted and entered into the causal control of action. This theoretical analysis, with elaborations, has also been extended to the clinical domain (Wilson and Dulany, 1983), and by others with variations to social psychology. The work was an opening—and in fact among the many studies that contributed to the overturn of behaviorism and the “cognitive revolution” of the 1960s and 1970s.

This was one way to address the “grander questions” that have motivated my research: the experiments provide scientific evidence for a theory of causally active volitional states, and conscious states that symbolically represent a reality beyond themselves.

## Cognitivism And Challenges

The cognitive revolution was a return to a science of mind—but it was, disappointingly to some, a continuation of the de-emphasis on a role for consciousness. Cognitivism is really a metatheory, computational and information processing views of mind deeply guided by the computer metaphor, the kind of mechanical model that some needed for sufficient confidence that mind was material enough after all to be a subject for science. On the computational view of mind, cognition runs in the brain the way software runs in the hardware of a computer, the famous “basic analogy.” Furthermore, mind is thought of as having three levels: i) a level of the brain processes; ii) a level of cognition as a sequence of causes and effects—the “real” mental activity; iii) a level of consciousness as an epiphenomenon, an only occasional emergent with no causal effects—a helpless rider. On the information processing view, computers and minds have also been thought to have analogous structures: sensory stores like input registers, long term memories like hard drives, action systems like output-registers, and a working memory like RAM (random access memory), something with a small sub-system for attention—with which consciousness was skittishly identified.

Outside that small focus of conscious attention, this metatheory holds that symbolic representations may be carried out unconsciously in all the other subsystems, even prior to conscious attention and outside focused attention in working memory for high level thinking, although all these claims have been experimentally challenged. Dennett (1987) put it simply: “So far as cognitive science is concerned, the important phenomena are the unconscious mental representations” (p. 218). And Kihlstrom (1987) published an influential paper in *Science* entitled “The cognitive unconscious” —a conception of the unconscious I have sometimes described as the “psychoanalytic unconscious expurgated.” It had initial plausibility with residues of the psychoanalytic unconscious rather deeply ingrained in the culture. (Only a few months ago, March 27,_2006, the full cover of *Newsweek* magazine presented Freud's picture with the heading, “Freud is NOT dead.” See *http://www.msnbc.msn.com/id/12112967/site/ newsweek/* for cover page.)

### Causal Reasoning

But what about something as complex and deliberative as causal reasoning—or “diagnostic reasoning” as we have also referred to it? We might think of it as involving hypotheses, evidence, and prior suppositions, all conscious states symbolically representing events beyond themselves. But in context of the standard cognitive metatheory, the prevailing theories were those in which one or another algorithm was said to describe the association of presence and absence of potential causes with presence and absence of an effect—with consciousness only incidental and usually unmentioned in the formal analysis. In a series of experiments (Carlson and Dulany, 1988), we examined an alternative theory I had described earlier, which can be made more concrete by thinking of what our subjects had to think about in these experiments. Sitting before a computer screen, they viewed the description of a murder scene, with Sir Charles the victim at his estate with weekend guests. On each trial, subjects viewed a clue said to be associated or not associated with each of two among the several suspects, then reported (by computer entry) several consciously represented propositions: prior degree of belief in guilt or innocence of each of those two suspects (the possible causes), a prior conscious supposition as to the incriminating or exonerating weight of the clue, their degree of belief that this evidence was incriminating or exonerating for each suspect. Then on the next trial, they again reported their degree belief in the guilt or innocence of each suspect, and then the other beliefs as well. The theory describes deliberative inferences in which prior belief in causal hypotheses is revised in light of the consciously represented evidence and suppositions, and the linear difference equations describing the interrelation among those belief states strongly predicted revision of belief in those causal hypotheses.

The grander questions? Notice that these conscious states symbolically represent events beyond themselves, and the experiments provide still another demonstration of how questions about a causal role for consciousness can be addressed with scientific methods.

### Category Learning

Here is another question about a role for consciousness, one that has been actively investigated, and one in which I became actively involved with research that was the first to challenge the standard view (Dulany, Carlson and Dewey, 1984; Dulany, 1997). Suppose we are learning to categorize any of a range of complex events, actions, or objects out there in the world—a very common and significant kind of learning. For experimental precision and control, the essence of this learning has frequently been represented in a simple little experimental task in which subjects learn to categorize strings of letters, letters which alone and in combination have various validities for predicting the categorization of the string. For one categorization, the letters also follow a finite state grammar. Can we unconsciously abstract rules—rules following that grammar or relating those letters to one or the other categorization? And can unconsciously represented rules then guide our categorizations? That is the standard view of “unconscious implicit learning”.

Alternatively we may learn to categorize in one of these two ways in which conscious states play a central and essential role. In “explicit learning” we venture consciously represented hypotheses, and strengthen or revise them in the light of consciously represented evidence, then act on consciously represented rules (a variant of the theory of causal learning described above). In “implicit learning”, however, we establish simpler associative relations between a conscious sense of certain letters in a string and a conscious sense of category membership. This is an associative relation which can sometimes be represented in consciously represented rules (something termed “higher order awareness.”). Our experiments over the years have identified conditions for both kinds of learning, learning in which validities of the rules reported predict the categorization without significant residual, leaving no room for the use of unconsciously represented rules.

Once more these are experiments addressing those “grander questions,” providing evidence for conscious mental states having causal and symbolic roles, evidence also inconsistent with those same roles being carried out by unconscious mental states.

Nevertheless, I must say that the literature of implicit learning continues to be experimentally active—and controversial. In fact, it is one of the two literatures in which there is still the most active experimental examination of claims for unconscious symbolic representations, some being supportive and others providing methodological criticism and support for alternative theory. The second is the literature examining claims for unconscious perception, with masking or brief exposure of stimuli for normal subjects, and in prosopagnosia and “blindsight” in patients with brain damage. Methodological critiques of claims for unconscious perception in these paradigms can be found in various sources, including Dulany (2001, 2004).

## Mentalism

I have felt it important to examine how answers to these deeper questions about the mind have shifted from early attempts to establish a science of consciousness, through behaviorism, and then cognitivism. I have also tried to lay out a mentalistic metatheory embracing the theories I have offered as well as consistent with the experimental work we and others have done. And more on all of this than can be presented here could be found in Dulany (1991, 1997, 2001, 2003, 2004, in press) and other sources. The mentalistic metatheory addresses those deeper questions, offering an alternative to the behavioristic and cognitive metatheories. It would be another science with consciousness central to its subject matter, but conceptually different than the 19^th^sub century attempts and recognizing what is non-conscious.

Put simply, with consciousness we symbolically represent the present in perception, the past in remembrance, and the future in intentions, expectations, hopes, and fears, etc. Anything remote from us in the present, past, or future can only enter thought symbolically. And symbols may be functionally specified by their ability to activate other symbols, appear as subjects or predicates in propositions, and be warranted by acting on those thoughts. Thinking “coffee cup” and picking it up warrants that symbol. In higher order awareness we can even symbolically represent our own conscious states—as I just did. On this view, consciousness evolved to provide the adaptive value of symbolic representation, and uniquely so. Very importantly, those conscious states causally influence other conscious states and actions.

For psychological science, we also need to view consciousness more analytically than as an attentional “system.” *Conscious states* come in familiar modes, each varying quantitatively—beliefs, percepts, intentions, expectations, specific feelings, etc. These conscious modes carry specific contents: belief that___, fear of ___, and those contents can have quantitative relations to objective events out there in the world. Furthermore, these conscious states are held with a degree of possession as “mine”, varying from moment to moment, something that may even diminish in certain neurotic states and be missing throughout a psychotic episode. Thus for theoretical and experimental analyses we can think of a conscious state as at the intersection of a set of measurable variables.

Our *mental episodes* come in two fundamentally different types:

1. Some mental episodes are *deliberative*, such as inferences and decisions, consisting of non-conscious mental operations that interrelate consciously held propositional contents. These propositional contents are carried by propositional *modes*, and in the example of “belief” we express this as “I (believe) *that* _____, “for example, “I believe that the temperature is unusually hot today.” This could then be combined with another propositional state in an inference or decision.
2. Other mental episodes are termed *evocative,* or *associative-activational*, where non-conscious activation interrelates sub-propositional contents carried by sub-propositional modes of awareness. We may have only a sub-propositional “awareness *of* ___ “which activates another “awareness *of* ___”, for example a sense of dinner cooking which activates a sense of hunger. Furthermore, these sub-propositional contents may be a conscious identification of something as such—a person or thing, or anything else at the immediate focus of attention. But they may also be only “literal” and outside or prior to attentional focus, as for example the orchestral surround of a lead instrument or the sound of a voice for a moment or two before we wrench attention from reading and identify what was said. If conditions are appropriate, we may also symbolically represent either kind of mental episode in *higher order awareness*.

What is non-conscious? In addition to those non-conscious mental operations, which are simply the brain processes interlinking conscious states, all these mental episodes occur between non-conscious sensory transducers and motor transducers. And what we know but are not thinking of at the moment is of course out of awareness, but in a non-symbolic neural network established in learning and activated in remembering. Although the term “unconscious” has traditionally been used for symbolic states and mental episodes said to be out of awareness, the term “non-conscious” is used here, and elsewhere, to refer to what is outside awareness but lacking those higher functions.

## Concluding Remarks

How then do I think a mentalistic psychology could most productively proceed? It is important for private reports to be used—without, however, a return to the 19^th^ century introspectionists’ exhaustive and exhausting analysis of conscious contents into irreducible elements, something beyond the limits of memory, vocabulary, and patience of ordinary mortals. It is important that conditions for valid reporting be maximized, as they should be for any measurement in science, in this case by observing those limits. It is also important for reporting to be constrained by theory guided questions and scales.

Where doubts remain about any assessment, investigators should call upon the logic of theoretical networks within the philosophy of science. When theoretical assertions and assessment assertions are part of the same theoretical network and predict results that are found, we gain confidence in both the theory and the validity of the assessments. With the richness of the network we also gain competitive support over other interpretations.

There would then be the theoretical and experimental challenges of re-examining much within the wide range of human experience that has been the subject of psychology.

I do recognize that there are many significant contributions in psychology that proceed apart from what *I* have seen as the “grander questions” for understanding the nature of the mind—and these, too, are of course part of what psychology has meant to me as a professor of psychology and journal editor. But all of that would be too extensive for this brief article, which I have been happy to write as a rather self-centered “What Psychology Means to *Me.”*^20^

## Questions That This Paper Raises*

These are questions not considered above, although some have been considered in the literature and are subject to further examination:

1. Can automaticity, despite its loose identification with “unconscious” in the vernacular, be covered by this metatheory? Any mental episode, evocative or deliberative, embodies automaticity in the sense that once started it is run off without intention—although we may have *prior* intentions to infer, decide, etc. The process of automatizing a complex action—for example, learning to drive with a hand shift—can also be explained, despite early cognitive theory that initial intentions for each part of the act drop *down* to an unconscious level. On an analysis in Dulany (1997), intentions drop *out* not *down* to an unconscious, as simpler activational connections are associatively established within a controlling neural network. This is supported by accumulating evidence that brain-imaging shows decreased activation in relevant areas with automatization. This general view is explicitly employed by Tzelgov (1997).
2. Are the clinical insights of dynamic psychology, those that have led to saying that a patient-client's action is controlled by something unconscious, reconcilable with the mentalistic metatheory? For example, could repression be viewed as a volitionally controlled rejection of a painful remembrance, as in Freud's (1892) early writing, or an anxiety-reducing substitution of thought directed elsewhere, as in Freud's (1926) later writing? Would there be a need for an unconscious casting of the objectionable to an inaccessible but dynamic unconscious, as in much of Freud's other writing and a general understanding? Perhaps these conceptions of repression would be more consistent with accumulating evidence that painful experiences increase amygdala activation and *enhance accessibility* of painful memories—when not voluntarily or habitually avoided. For that matter, could acquisition of defenses and some symptoms be viewed as forms of implicit learning or explicit learning?
3. Could many instances usually thought of as unconsciously controlled action be explained by activation from cues in consciousness and a non-symbolic neural network in the absence of the subject-patient-client's ability to represent the significant prior mental episodes in higher order awareness? Memory and powers of inference are limited, especially with lapses of time from the significant earlier events. But theory of this origin is the theorist's business not theirs.
4. Within the social domain, are there prejudiced actions toward a minority member or group that could be explained in the same way, in spite of the actor's defensive claims that the action was not prejudiced?
5. Under what circumstances can persons have intentional control over their own *thoughts?* This constitutes a very interesting question of the ability to represent *future* thought in higher order awareness and successfully act on it. It is a question that has been under investigation by Carlson (2002) and others, making use of aspects of this metatheory.
6. We can also ask to what degree acquisition of grammar could be explained by an associative activational process, a process extensively investigated and modeled, by Perruchet and Vinter (2002), who also explicitly employ aspects of this metatheory. We could welcome an alternative that avoids theoretically asserting that the grammar in the consciousness of the sophisticated linguist is also the grammar in the unconscious of the unsophisticated speaker-listener.
7. How adequately could this metatheory represent what is, in a very general way, referred to as the Self? The sense of possession of one's own mental states that may vary in strength? And the conscious beliefs we have about our past, present, and future abilities, feelings, and actions? These are all propositional representations with “I” or “Me” as subject. And should we see the “traits” of personality theory simply as abstract constructs—inferred from sets of consciously endorsed self-propositions on personality tests?

## About the Author

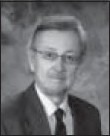



**Donelson Dulany** is currently Professor of Psychology Emeritus at the University of Illinois and Editor, American Journal of Psychology. He received his PhD from the University of Michigan, and his AB from the University of Tennessee. After his PhD and two years in the US Army, with the Department of the Army Liaison and Research Office, he joined the faculty of the Department of Psychology at the University of Illinois. He has co-taught a summer graduate seminar at Harvard University. He is a member of the Psychonomic Society, the Association for the Scientific Study of Consciousness, Fellow of the American Psychological Association, Fellow of the Association for Psychological Science, and listed in Who's Who in America.

## Footnotes

* **Editor's note:** Placed in the body of the text on request by the author, since it is referenced to many other researchers in the field.

## References

[1] Carlson RA, Ross B (2002). Conscious intentions in the control of skilled mental activity. Learning and Motivation.

[2] Carlson R, Dulany D (1988). Diagnostic reasoning with circumstantial evidence, Cognitive. Psychology.

[3] Dennett D (1987). The Intentional Stance.

[4] Dulany DE, Dixon T, Horton D (1968). Awareness, rules, and propositional control: A confrontation with S-R behavior theory. Verbal Behavior and General Behavior Theory.

[5] Dulany DE, Carlson RA, Dewey GI (1984). A case of syntactic learning and judgment: How conscious and how abstract?. Journal of Experimental Psychology: General.

[6] Dulany DE, Wyer RS, Srull TK (1991). Conscious representation and thought systems. Advances in social cognition.

[7] Dulany DE, Cohen J, Schooler J (1997). Consciousness in the explicit (deliberative) and implicit (evocative). Scientific approaches to consciousness.

[8] Dulany DE (2001). Inattentional awareness. Psyche: An Interdisciplinary Journal of Research on Consciousness.

[9] Dulany DE (2003). Strategies for putting consciousness in its place. Journal of Consciousness Studies.

[10] Dulany DE, Gennaro RJ (2004). Higher order representation in a mentalistic metatheory. Higher order thought theories of consciousness.

[11] Dulany DE Psychology and the study of consciousness. Oxford Companion to Consciousness.

[12] Freud S, Strachey J (1896). On the theory of hysterical attacks. The standard edition of the complete psychological works of Sigmund Freud.

[13] Freud S, Strachey J (1926). Inhibitions, symptoms, and anxiety. The standard edition of the complete psychological works of Sigmund Freud.

[14] James W (1890). Principles of psychology.

[15] Kihlstrom JF (1987). The cognitive unconscious. Science.

[16] Perruchet P, Vinter A (2002). The self-organizing consciousness. Behavioral and Brain Science.

[17] Titchener EB (1898). The postulates of a structural psychology. Psychological Review.

[18] Tzelgov J, Wyer RS, Srull TS (1997). Automatic but conscious: That is how we act most of the time. Advances in social cognition.

[19] Watson JB (1924). Behaviorism.

[20] Wilson MW, Dulany DE (1983). An analysis of cognitive control of self-disclosure in a clinical analogue. Cognitive Therapy and Research.

